# Serotype Distribution of *Salmonella* Isolates from Turkey Ground Meat and Meat Parts

**DOI:** 10.1155/2013/281591

**Published:** 2013-07-10

**Authors:** Irfan Erol, Muammer Goncuoglu, Naim Deniz Ayaz, Lüppo Ellerbroek, Fatma Seda Bilir Ormanci, Ozlem Iseri Kangal

**Affiliations:** ^1^Ankara University, Faculty of Veterinary Medicine, Department of Food Hygiene and Technology, 06110 Diskapi, Ankara, Turkey; ^2^Federal Institute for Risk Assessment (BfR) FGr 42-NRL for Campylobacter, Diedersdorfer Weg 1, 12277 Berlin, Germany

## Abstract

The aim of the study was to find out the serotype distribution of 169 *Salmonella* colonies recovered from 112 *Salmonella* positive ground turkey (115 colonies) and 52 turkey meat parts (54 colonies). Out of 15 *Salmonella* serotypes: *S.* Corvallis, *S.* Kentucky, *S.* Bredeney, *S.* Virchow, *S.* Saintpaul and *S.* Agona were identified as the predominant serovars at the rates of 27%, 13%, 12%, 12%, 11%, and 10%, respectively. Other serotypes were below 6% of the total isolates. All *S.* Kentucky and *S.* Virchow and most of the *S.* Corvallis (39/46) and *S.* Heidelberg (9/9) serotypes were recovered from ground turkey. The results indicate that turkey ground meat and meat parts were contaminated with quite distinct *Salmonella* serotypes. This is the first study reporting *Salmonella* serotype distribution in turkey meat and *S.* Corvallis as predominant serotype in poultry meat in Turkey.

## 1. Introduction

*Salmonella* is one of the major food-borne pathogens and has an importance as a leading cause of food-borne bacterial diseases in humans throughout the world [1]. According to the Centers for Disease Control and Prevention (CDC), *Salmonella* spp. are causing 1.4 million food-borne illnesses, 15,000 hospitalizations, and 400 deaths annually in the United States [2]. The total costs of food-borne *Salmonella* infections of humans in the US have been estimated to 3.3 billion dollars per year [3]. According to the European Surveillance System 2010 data, total of 99,020 confirmed *Salmonella* cases were reported through 27 European Union member countries with a notification rate of 21.5 cases per 100,000 population [4]. Different studies indicate that, foods of animal origin, particularly poultry, cattle and pig are the major vehicles of diseases caused by food-borne pathogens. Among them turkey meat and products are attributed to be the important sources of food-borne salmonellosis [4].

Poultry processing plays an important role to increase the contamination rate of *Salmonella* in turkey meat [5, 6]. Scalding, defeathering, evisceration and cooling steps in slaughtering are the critical points in contamination of carcass [5]. Also contamination of foods with this bacterium can occur at different processing line including distribution, marketing, handling and preparation both in processing plant or home. Therefore, turkey meat can easily be contaminated with *Salmonella* throughout the whole production chain [5, 7]. Nevertheless, *Salmonella* contamination in turkey flocks is generally asymptomatic and detection of the bacterium emerges by the randomly monitoring by the industry [6]. It was pointed out that serotype profiles of *Salmonella* in animal carcasses match with corresponding raw ground products [8] and findings of studies strengthened possibility of transmission of *Salmonella* to humans through the food chain [9].

The prevalence of *Salmonella* in turkey meat has been studied mainly in developed countries. The results of these studies showed that prevalence and serotype distribution of *Salmonella* was varied. The percentage of positive samples varied from zero to 40% in fresh turkey meat and higher than 5% in RTE turkey meat products in EU countries, the US and the Canada [5, 10–14]. The results of these studies showed that serotype profiles of the isolates were different.

Ground meat has high nutritional value and is useful for preparing foods. However, it is a suitable medium for growth of many pathogen and saprophyte microorganisms. Even if ground meat is originally contaminated at a low level with *Salmonella*, growth and/or cross-contamination may occur during storage and handling under poor hygienic conditions [15].

There are no published scientific data on serotype distribution of *Salmonella* spp. in turkey meat in Turkey. Therefore, this study aimed to determine the serotype distribution of *Salmonella* isolated from fresh turkey ground meat and meat parts obtained from retail markets in Turkey, to provide some scientific data for further epidemiological studies.

## 2. Material and Methods

### 2.1. Bacterial Strains

A total of 169 *Salmonella* spp. isolated from ground turkey and meat parts (meat cuts, breasts, and legs) purchased from different supermarkets located in Ankara between July 2004 and January 2006 were serotyped. Hundred and fifteen of the colonies were belonging to 112 *Salmonella* positive ground turkey meat samples that have been isolated in a previous study [16]. Additionally 54 colonies were obtained from 52 *Salmonella* spp. detected turkey meat part samples.

### 2.2. Isolation and Identification of *Salmonella* spp. 

For the isolation of *Salmonella* spp. standard cultivation technique was performed according to the Bacteriological Analytical Manual of the Food and Drug Administration [16, 17] and the International Organization for Standardization (ISO-6579) [18]. Twenty five grams of each sample was weighed into 225 mL of buffered peptone water (BPW, Oxoid CM0509, Hampshire, UK) for preenriching and shaken for about 2 min, then incubated at 37°C overnight. After incubation, 0.1, 1 and 1 mL of the preenrichment broths were added to 10 mL of Rappaport-Vassiliadis Enrichment broth (RV, Oxoid, CM669), 9 mL of Selenite Cystine broth (SC, Difco 112534 JC, Detroit, USA) and 9 mL of Tetrathionate (TT) broth (FDA, BAM), respectively. For selective enrichment, RV broth, SC broth at and TT broth were incubated at 43°C, 37°C and 42°C for 24 h, respectively. After incubation period broths were streaked on to Brilliant-green Phenol-Red Lactose Sucrose agar (BPLS, Merck 1.07237, Darmstadt, Germany) and Xylose Lysine Deoxycholate (XLD) (Merck 1.05287) agar and incubated at 37°C for 18–24 h. Up to five typical grown colonies were picked and inoculated into Triple Sugar Iron Agar (TSIA, Oxoid, CM0277), Lysine Iron Agar (LIA, Oxoid CM0381), and Urea Broth Base (Oxoid, CM0071B) and incubated at 37°C for 24–48 h. TSIA positive, LIA positive, and urease negative colonies were considered as suspect for *Salmonella*. The agglutination test was done with Polyvalent *Salmonella* Antisera (Difco, Cat. No L840114-1, Detroit, USA). Suspect *Salmonella* colonies were mixed with a drop of antiserum on a slide. Agglutination with antiserum was accepted as a positive reaction for *Salmonella* spp. isolates that showed agglutination were stored at 4°C on Tryptone Soya agar (TSA, Oxoid CM0131) and at −86°C (Sanyo MDF-U5186S, Japan) in cryovials for the PCR verification and serotyping.

### 2.3. PCR Confirmation of the *Salmonella* Isolates

In order to determine the origin of DNA replication *oriC* gene of *Salmonella* strains for the verification, PCR analysis were performed. For the PCR analysis *Salmonella* Typhimurium ATCC 14028 was used as positive control.

*DNA Extraction.* Isolates stored at 4°C in Tryptone Soy Agar (TSA, Oxoid CM 131) were incubated in Brain Heart Infusion broth (BHI, Oxoid CM0225) at 37°C for 24 h. Then 1 mL of each culture was separately transferred to microcentrifuge tubes. All tubes were centrifuged (Eppendorf Centrifuge 5417R, Hamburg, Germany) for 15 min at 5000 rcf at 10°C. The pellets were resuspended in 1 mL sterile aquabidest. The suspensions were mixed by vortex (IKA MS1 Minishaker, Wilmington, USA). Then all tubes were centrifuged for 5 min at 5000 rcf at 10°C. The pellets were resuspended with 200 *μ*L sterile aquabidest and incubated for 20 min at 95°C in a water bath (Memmert WB/OB 7–45, WBU 45, Schwabach, Germany) then cooled on ice.

*PCR Analysis for the Detection of oriC Gene.* The primers used are specific to the origin of DNA replication (*oriC*) on the *Salmonella* chromosome and produce a 163 bp DNA fragment (*Primer* 1: 5′-TTA TTA GGA TCG CGC CAG GC-3′, *Primer* 2: 5′-AAA GAA TAA CCG TTG TTC AC-3′). PCR was performed with a final volume of 50 *µ*L reaction mixture containing incomplete 5x PCR Buffer (Promega M7921, Madison USA), 1.5 mM MgCl_2_ (Promega A3511), 200 mM each of the deoxynucleoside triphosphates (dNTPs, Promega U1420), 1 U Taq DNA polymerase (Promega M3005), 0.50 mM each of primer and 10 *µ*L DNA. Thermal cycling (Biometra Personal Cycler, Goettingen, Germany) was carried out with the initial denaturation at 94°C for 1 min and then 35 cycles of denaturation at 94°C for 1 min, annealing at 53°C for 1 min, and extension at 72°C for 1 min, 72°C, 10 min for final extension [19–21]. A 10 *µ*L aliquot of each PCR product was subjected to 1.5% agarose gel electrophoresis containing 0.1 mg/mL ethidium bromide for 1 h at 100 V (Biometra, Agagel, B15339). Amplicon visualisation and documentation was performed using gel documentation and analysis system (Syngene Ingenius, Cambridge, UK).

### 2.4. Serotyping

Serotyping of the *Salmonella* isolates was performed at the National Reference Centre for *Salmonella* and other bacterial enteric pathogens, Robert Koch Institute (RKI-Wernigerode) with the scheme of Kaufmann-White using lam agglutination and serum neutralization tests [22].

## 3. Results

Conventional cultivation technique was used for the isolation of *Salmonella* spp. from turkey ground meat and meat parts. All 169 colonies (112 from ground turkey and 54 from turkey meat parts) were confirmed with PCR by detection of *oriC* gene. Using lam agglutination and serum neutralization tests, 15 different serotypes were identified among 169 *Salmonella* isolates. *S.* Corvallis (*n*: 46, 27%), *S.* Kentucky (*n*: 22, 13%), *S.* Bredeney (*n*: 20, 12%), *S*. Virchow (*n*: 20, 12%), *S*. Saintpaul (*n*: 18, 11%), and *S*. Agona (*n*: 17, 10%) were the most frequently isolated serotypes (Table 1). All 22 isolates of *S.* Kentucky and 20 isolates of *S*. Virchow were recovered from ground turkey meat. Likewise *S*. Heidelberg, *S*. Stanleyville, *S*. Montevideo, *S*. *subsp. I*, *S*. *group C*, and *S*. Newport were only recovered from ground turkey meat samples. As the predominant serotype 39 of 46 isolates of *S.* Corvallis were recovered from ground turkey meat. However other two major serotypes, 90% of *S.* Bredeney and 67% of *S*. Saintpaul isolates were recovered from turkey meat parts. Also, *S*. Hadar, *S*. *munchen* and *S*. Typhimurium were only recovered from turkey meat parts samples (Table 1), although only at low numbers.

The seasonal distribution of *Salmonella* serotypes was determined as follows: 39 (23%), 26 (15%), 53 (31%), and 51 isolates (30%) during winter, spring, summer and autumn, respectively (Table 2). Most of the isolates were determined in warm months. In winter and spring 16 different serotypes, in summer and autumn 19 different serotypes were recovered. As a predominant serotype *S.* Corvallis was detected in spring, summer, autumn and second after *S.* Bredeney in winter months. Also, 86% of *S.* Kentucky, as the second major serotype of the study, was recovered in spring, summer and autumn.

Ground turkey meat samples were collected from nine different companies and no correlation was observed between producing companies and serotype profiles. Unlikely, meat part samples were collected from three different supermarkets (supermarkets A, B and C) and these meat parts were prepared in their own butchery stores. Based on the results of this study a relation as observed between supermarkets and serotype distribution of samples. As shown in Table 1, *S.* Bredeney and *S*. Saintpaul were the mostly recovered serotypes from turkey meat parts. Within the 78% of *S*. Bredeney and 58% of meat parts recoveries of *S*. Saintpaul serotypes were detected in supermarket A.

## 4. Discussion

In this study, out of 169 *Salmonella* turkey meat isolates 15 different serotypes were identified and showed quite distinct distribution. Among them *S.* Corvallis was found to be the predominant serotype. In a study, *S.* Kentucky, *S*. Anatum and *S*. Heidelberg were reported as the most frequently isolated serotypes from turkeys and their environments [23]. In Great Britain, *S*. Kottbus, *S*. Kedougou, *S*. Derby, *S*. Senftenberg, *S*. Newport and *S*. Oslo were the most common serotypes recovered from different types of turkey flocks [9]. *S*. Typhimurium, *S*. Newport, *S*. Derby, *S*. Indiana and *S*. Agona were the top five reported serovars in British turkey flocks between 1995 and 2006 [24]. According to the EFSA Scientific Report *S*. Bredeney, *S*. Hadar, *S*. Derby, *S*. Saintpaul, *S*. Kottbus and *S*. Typhimurium were the most frequently isolated serotypes in turkey flocks [25]. The contamination of poultry meat by different *Salmonella* serotypes is straightly contact with the flock contamination by the slaughtering process and the contamination of carcasses, and meat mainly during evisceration, also the link between levels of *Salmonella* in the flocks and the slaughterhouse are well defined [8, 26]. Further researches should focus on the controlling the sources of contamination with *Salmonella* spp. and serotypes in turkey meat industry.

There were several reports about distribution of *Salmonella* serotypes in raw turkey meat samples. In turkey meat in Germany, *S*. Saintpaul, *S*. 1,4,(5),12:i:- and *S*. Newport were identified as the major serotypes [27]. A previous study conducted in Canada reported that *S*. Heidelberg and *S*. Hadar were the most common serotypes recovered from 91 turkey meat samples including drumstick, wing or ground turkey [14]. In a different study, *S*. Newport, *S*. Hadar, *S*. Heidelberg, *S*. 4:12: nonmotile and *S*. Reading were recovered from retail turkey meat samples in North Dakota, USA [10]. Khaitsa et al. [12] reported six *Salmonella* serotypes from 959 turkey products as follows: Hadar, Heidelberg, Typhimurium var. Copenhagen, Newport, Saintpaul and Agona. Most of the *Salmonella* serotypes recovered during this study are common isolates from turkey meat samples. Like previous reports, in this study *S*. Enteritidis and *S*. Typhimurium were not considered as the most frequently encountered serotypes in turkey meat samples [28]. However Beli et al. [29] reported *S*. Enteritidis as the major serotype isolated from turkey meat samples. Similar results arise about specific serotypes within other studies. A study from the USA has reported that *S*. Saintpaul was almost specific for turkey meat rather than chicken, beef and pork samples [11]. Although the serotype of concern was detected with 11% in turkey meat samples in this study, the results of another work conducted in our country showed that *S*. Saintpaul was not detected in any of broiler carcass and edible offal samples in 70 *Salmonella* positive isolates [30]. The number of turkey farms in our country is relatively low, as well as the number of turkey meat production plants. Therefore presence of a particular serotype in these limited number of facilities may cause a serovar as the most detected one over 2500 *Salmonella* serotypes. In addition serotype variation among these studies is probably due to contaminated feed, infected breeding flocks [24], differences in production system of turkey meat and contamination of meat at slaughterhouse process [8], variability in sampling of isolates from different sources [31], geographical features, socioeconomic and cultural differences between countries, national or international control and surveillance program differences, and also some yearly variations [32, 33].

There have been numerous reports of human salmonellosis due to consumption of different turkey products by different *Salmonella* serotypes including *S*. Reading, *S*. Hadar, *S*. Agona, *S.* Saintpaul, and *S*. Typhimurium [34–38]. All the *Salmonella* serotypes recovered from this study except serotype Reading were common in reported international human salmonellosis cases that were caused by consumption of contaminated turkey meat [39–41]. However these serotypes frequently recovered in this study have never been reported as an agent of human salmonellosis in Turkey. In the EU *S.* Corvallis and *S.* Kentucky, which are first two major isolates of this study, were reported from human salmonellosis cases [42, 43].

In conclusion, turkey meat can be contaminated with quite distinct serotypes. According to our results fifteen different *Salmonella* serotype were recovered and among them *S.* Corvallis was detected as a predominant serovar. These results showed that upcoming *Salmonella* monitoring programme should cover turkey meat production chain in Turkey.

## Figures and Tables

**Table 1:** Number and percentage of *Salmonella* serotypes isolated from ground turkey and turkey meat parts.

Serotypes	Percent	Number of isolates				Total
		Ground turkey	Meat cuts	Breasts	Legs	
*S.* Corvallis	27	39	5	1	1	**46**
*S.* Kentucky	13	22	—	—	—	**22**
*S.* Bredeney	12	2	6	2	10	**20**
*S.* Virchow	12	20	—	—	—	**20**
*S.* Saintpaul	11	6	6	—	6	**18**
*S.* Agona	10	9	3	3	2	**17**
*S.* Heidelberg	5	9	—	—	—	**9**
*S.* Hadar	3	—	2	2	1	**5**
*S.* Munchen	2	—	—	2	1	**3**
*S.* Stanleyville	1	2	—	—	—	**2**
*S.* Montevideo	1	2	—	—	—	**2**
*S. subsp. I *	1	2	—	—	—	**2**
*S. group C *	1	1	—	—	—	**1**
*S.* Typhimurium	1	—	1	—	—	**1**
*S.* Newport	1	1	—	—	—	**1**
Total		**115**	**23**	**10**	**21**	**169**

**Table 2:** Seasonal distribution of *Salmonella* serotypes.

Serotypes	Winter	Spring	Summer	Autumn	Total
*S.* Corvallis	8	11	14	13	**46**
*S.* Kentucky	3	9	7	3	**22**
*S.* Bredeney	10	—	10	—	**20**
*S.* Virchow	—	1	1	18	**20**
*S.* Saintpaul	4	3	7	4	**18**
*S.* Agona	4	1	1	11	**17**
*S.* Heidelberg	2	—	7	—	**9**
*S.* Hadar	4	—	—	1	**5**
*S.* Munchen	2	—	—	1	**3**
*S.* Stanleyville	1	—	1	—	**2**
*S.* Montevideo	—	—	2	—	**2**
*S. subsp. I *	1	1	—	—	**2**
*S. group C *	—	—	1	—	**1**
*S.* Typhimurium	—	—	1	—	**1**
*S.* Newport	—	—	1	—	**1**
Total	**39**	**26**	**53**	**51**	**169**
